# Smoking related attention alteration in chronic obstructive pulmonary disease-smoking comorbidity

**DOI:** 10.1186/s12890-022-01964-6

**Published:** 2022-05-06

**Authors:** Feiyan Zeng, Wei Hong, Rujing Zha, Ying Li, Chen Jin, Ying Liu, Hao Liu, Mengqiu Liu, Mei Liu, Fei Xu, Daiju Hu, Hongwen Song, Haiyan Wu, Yongqiang Yu, Xiaochu Zhang

**Affiliations:** 1 Department of Radiology, the First Affiliated Hospital of USTC, Department of Psychology, School of Humanities & Social Science, Division of Life Science and Medicine, University of Science & Technology of China, Hefei, 230027 China; 2 School of Earth and Space Science, University of Science & Technology of China, Hefei, 230027 China; 3 Application Technology Center of Physical Therapy to Brain Disorders, Institute of Advanced Technology, University of Science & Technology of China, Hefei, 230031 China; 4 Hefei Medical Research Center on Alcohol Addiction, Affiliated Psychological Hospital of Anhui Medical University, Hefei Fourth People’s Hospital, Anhui Mental Health Center, Hefei, 230017 China; 5 Centre for Cognitive and Brain Sciences (CCBS), University of Macau (UM), Macau SAR, China; 6 Department of Radiology, The First Affiliated Hospital of Anhui Medical University, Hefei, 230022 China; 7 Biomedical Sciences and Health Laboratory of Anhui Province, University of Science & Technology of China, Hefei, 230027 China

**Keywords:** Chronic obstructive pulmonary disease (COPD), Smoking, Comorbidity, Nicotine, Attention ability, Functional magnetic resonance imaging (fMRI)

## Abstract

**Background:**

Chronic obstructive pulmonary disease (COPD) is a respiratory disease that causes a wide range of cognitive impairments. Although COPD-Smoking comorbidity is common, the relationship between smoking and cognitive function in COPD-Smoking comorbidity remains unclear. This study aimed to investigate the effect of smoking on cognitive function like attention in COPD-Smoking patients.

**Methods:**

In this study, we used the Montreal Cognitive Assessment (MoCA) scale and resting-state functional magnetic resonance imaging (fMRI) to explore the effect of smoking on attention in patients with COPD.

**Results:**

Behavioral analysis revealed that among patients with COPD the smokers had a shorter course of COPD and showed a worse attention performance than the non-smokers. Resting-state fMRI analysis revealed that among patients with COPD smokers showed lower regional homogeneity (ReHo) value of the fusiform gyrus than non-smokers. Importantly, the ReHo of the fusiform gyrus is positively associated with attention and mediates the effect of smoking on attention in COPD.

**Conclusions:**

In summary, our study provides behavioral and neurobiological evidence supporting the positive effect of smoking on attention in COPD. This may be helpful for understanding and treating COPD and even other diseases comorbid with smoking.

**Supplementary Information:**

The online version contains supplementary material available at 10.1186/s12890-022-01964-6.

## Background

Chronic obstructive pulmonary disease (COPD) is a chronic respiratory disease and one of the leading causes of morbidity and mortality worldwide [1, 2]. It is characterized by persistent respiratory symptoms and limited airflow [3–5] and is an independent risk factor for cognitive impairment [6, 7]. Poor cognitive function in patients with COPD may result in a greater risk of hospitalization, longer hospital stays, and worsening health status, which may lead to an increased mortality rate [8, 9]. Therefore, it is of great clinical significance to explore the mechanism of impaired cognitive function in COPD patients.

Smoking has been recognized as the leading cause of COPD, as the long-term stimulation of cigarette smoke eventually leads to COPD [10, 11]. Studies have shown that approximately 80–90% of COPD patients have a history of smoking, and half of the smokers will eventually develop COPD [12]. Despite the prevalence of the comorbidity between COPD and smoking, the role of smoking in cognition and brain damage in COPD-Smoking comorbidity is largely unclear.

A line of studies has demonstrated the existence of cognitive impairments in COPD. A recent review found that COPD patients commonly exhibited deficits in attention, memory, executive function, psychomotor function, and language [13]. Specifically, Anna et al. compared former smokers with and without COPD and found that smokers with COPD had impairment in attention, characterized by slow processing, inattention, and impulsivity [14]. Nelly et al. found that patients with COPD had insufficient attentional resources for successfully dealing with dual tasks, which led to greater gait variability [15].

Another line of studies revealed abnormal brain structure and function in COPD patients. For example, Wang et al. found that the grey matter volume in the bilateral fusiform gyrus, bilateral calcarine, right superior temporal gyrus, right middle temporal gyrus, left precuneus and right inferior parietal lobule were significantly reduced in COPD patients compared with normal controls, and their forced vital capacity (FVC%) was closely related to the volume of cortical grey matter [16]. The lower gray matter volume of the above-mentioned regions may explain the lower ability of naming, memory, and visuospatial and executive function in COPD patients [16]. Using the functional connectivity analysis, Li et al. found that the connectivity density of the right lingual gyrus (LG), bilateral supplementary motor area (SMA), and right lateral lobules (PCL) were significantly reduced in COPD patients than that in normal controls [17]. Further seed-based functional connection analysis revealed that COPD patients had decreased functional connections in the left anterior cerebellar lobe, left fusiform gyrus, right insula lobe, right inferior frontal lobe, left putamen, and other brain regions.

As already mentioned, smoking can cause COPD [10]. A line of studies has shown that smoking itself caused attention disability. Domino et al. found that compared with non-smokers, smokers had an increased risk of cognitive impairment [18]. Smoking also leads to chronic attention maintenance deficits [19]. Another line of studies revealed abnormal brain structure and function in smokers. Due et al. found that in smokers, both reward and attention circuits were activated by exposure to smoking-related images including pictures of people smoking, hands holding cigarettes, and pictures of cigarettes alone. These cues were processed like addictive drugs in that they activate mesolimbic reward regions. Such reward stimulus may further increase the activation of the attentional regions such as the bilateral parietal lobe, and fusiform gyrus [20]. Konishi et al. also demonstrated that long-term smoking can cause damage to the prefrontal cortex, which leads to long-term attention maintenance regulation deficits [19]. In smokers, Jürgen et al. observed a significantly smaller grey matter volume and lower grey matter density in the anterior cingulate, prefrontal cortex, orbitofrontal cortex, occipital lobe, temporal lobe, and parahippocampal gyrus. The amount of tobacco smoke exposure was negatively correlated with the volume of the frontal lobe, temporal lobe, and cerebellum. Structural damage in the cortex and subcortical regions of smokers was associated with deficits in the brain networks of attention and working memory [21].

Although smoking leads to extensive damage to brain structures and function, nicotine neuroprotective effect has been found in numerous molecular and cellular biological studies [22]. Nicotine also has a protective effect on dopaminergic neurons. The activation of nicotinic acetylcholine receptors (nAChRs) will ultimately result in the release of dopamine as well as be implicated in the neural modulation of inflammation [22]. And the local administration of nicotine into the substantia nigra can appear the activation of the nAChRs, namely a4b2 and a7, on dopaminergic nerve terminals in the corpus striatum [23], And it was also well documented that the number of 3[H] nicotine binding sites, a4b2 nAChRS, are increased in the brains of smokers examined postmortem [24–26]. In addition to potential neuroprotective behaviors, from learning and memory enhancement to addiction and neuroprotection, nicotine also has antidepressant properties and the ability to improve cognitive activities such as attention [27] and cognitive control [28].

On the one hand, studies on the long-term effect of smoking showed that long-term smoking would impair attention ability [20]. On the other hand, research on the acute effect of smoking has shown that smoking could improve attention [22]. Despite the long-term effect of nicotine on attentional performance, we expected improvements in attentional performance in patients with comorbidity of smoking and COPD.

In the present study, we first explored the effect of smoking on attention in COPD patients with smoking comorbidity and located regions of interest (ROIs) whose regional homogeneity (ReHo) showed alterations in this population. Finally, we tested the relationship between these brain regions and smoking and attention ability.

## Methods

### Participants

From October 2014 to August 2018, 42 patients with COPD diagnosed in Anhui Provincial Hospital and followed up in an outpatient clinic were collected, including 20 COPD patients who smoked (COPD-Smoking group) and 22 non-smoking COPD patients (COPD-Nonsmoking group). During the same time, we collected 43 healthy subjects diagnosed as non-COPD by pulmonary function examination in Anhui Provincial Hospital, including 22 smokers (NonCOPD-Smoking group) and 21 non-smokers (NonCOPD-Nonsmoking group). Written informed consent was obtained from subjects before experiments.

### Inclusion and exclusion criteria

All the selected COPD patients met the global agreement diagnostic criteria [29] and had dyspnea, chronic cough or expectoration, etc. According to pulmonary function examination, forced expiratory volume in one second (FEV1%) < 70% after inhaling bronchodilator indicates an incompletely reversible airflow limitation. And the pulmonary function examination of the healthy subjects must be normal. None of the participants underwent oxygen therapy, and no acute attack had occurred in the past month. All smokers, including the participants in the COPD-Smoking group and the NonCOPD-Smoking group, had a smoking history ≥ 20 years, ≥ 5 cigarettes smoked a day and a smoking index ≥ 200. Both COPD-Nonsmoking and NonCOPD-Nonsmoking had never smoked before.

Participants with other respiratory diseases, obstructive sleep apnea, history of head trauma, intracerebral tumors and other uncured tumors, epilepsy, substance abuse, cardiovascular disease, diabetes, mental illness, Alzheimer's disease, sleep disorders, anemia, alcohol abuse, and MR contraindications were excluded. None of the smoking subjects had ever quit smoking. All participants in this study signed informed consent before the examination.

### Procedure

Every subject underwent a pulmonary function test, resting-state functional magnetic resonance imaging (fMRI) scan, cognitive function assessment, and demographic survey. The demographic survey collected information on age, sex, and years of education, the course of COPD (years); additionally, information on smoking history and smoking index was collected only for smokers (Table 1).

**Table 1:** Demographic characteristics of COPD and NonCOPD group

Characteristics	COPD(n = 42) Mean ± SD	NonCOPD (n = 43) Mean ± SD	*p*
Age	69.64 ± 8.00	68.30 ± 8.50	0.456
Gender (female/male)	14/28	13/30	0.759
Education	5.76 ± 4.79	5.77 ± 4.20	0.995
Smoking/nonsmoking	20/22	22/21	0.590
Smoking index	796.25 ± 391.80	745.68 ± 358.56	0.665
Smoking history	41.60 ± 9.36	38.82 ± 11.58	0.400
FEV1%	52.36 ± 17.05	102.14 ± 9.96	< 0.001
FVC%	65.91 ± 15.59	90.00 ± 6.97	< 0.001
FEV1/FVC	56.31 ± 8.83	87.14 ± 5.01	< 0.001

The smoking index was calculated by (years of smoking) * (number of cigarettes smoked per day). The smoking history was the years of smoking. FEV1%: forced expiratory volume in one second. FVC%: forced vital capacity

#### Pulmonary function assessment

All subjects were examined by an experienced pulmonary function examiner using the CareFusion Vmax Encore Autobox. The interval between the pulmonary function test and MRI scan was ≤ 2 days. The percentage of the FEV1% the percentage of FVC%, and FEV1/FVC were recorded to indicate pulmonary function, with smaller values indicating poorer pulmonary health.

#### Smoking behavior assessment

All smokers recorded the information on smoking behavior, including smoking history (years of smoking), the number of cigarettes smoked per day, and smoking index. The smoking index was calculated by (years of smoking) * (number of cigarettes smoked per day) [30]. The smoking index of all smokers is ≥ 200.

#### Cognitive function assessment

The MoCA scale [31] was used to measure visuospatial execution ability, naming ability, language ability, attention ability, abstraction ability, orientation ability, and delayed recall ability. All subjects filled out the MoCA Scale after fMRI scanning, which was completed within 10–15 min. Participants with a score of less than 26 on the MoCA scale were considered to have cognitive dysfunction; those with a score of ≥ 26 were considered to have a normal cognitive function; those with less than 12 years of education were given an additional point to their initial score.

#### Resting-state fMRI scan

For magnetic resonance data acquisition, a Siemens Trio 3.0 T superconducting MRI scanner with an 8-channel head coil was adopted to acquire data. Subjects were asked to lie supine, close their eyes and remain awake, while avoiding thinking and experiencing mood swings. (1) Anatomical T1 weighted images were acquired using 3D magnetization-prepared rapid gradient-echo sequence, repetition time/echo time (TR/TE) = 876/18 ms, slice thickness = 1.0 mm, slice number = 176, matrix = 224 × 256, FOV = 240 mm × 240 mm, flip angle = 55°. (2) Resting-state functional scans were acquired with spin-echo E-Planar imaging (SE-EPI) sequence, TR/TE = 2000/30 ms, slice thickness = 3.7 mm, slice number = 33, matrix = 64 × 64, FOV = 240 mm × 240 mm, flip angle = 85°. A total of 240 volumes were acquired, and the scanning time was 8 min and 6 s.

### Resting-state fMRI analysis

#### Preprocessing

Considering the instability of the instrument and the subjects at the beginning of the scanning, the first 10 TR of the scanning were excluded. Then slice time correction was performed at the remaining time points to reduce the heterogeneity of the data at different levels due to different acquisition times. The spatial correction was performed to correct for the effect of the head motion, any TR with head motion > 2.5 mm of translation or 2.0° of rotation during the scan was excluded. The resting-state functional image was registered to the structure image, and the space is normalized to the MNI standard space. For nuisance signal correction, the following nuisance parameters were included as regressors within the general linear model; 6 motion parameters and their first derivatives, white matter (WM), cerebrospinal fluid (CSF), and a linear trend term. Gaussian kernel (FWMH = 6 mm) was used for spatial smoothing to reduce the influence of spatial noise on scanning and improve the signal-to-noise ratio. Linear drift removal and filtering were performed. 0.01–0.1 Hz frequency band was adopted to filter out physiological noise such as breathing and heartbeat.

#### Functional connectivity

After previous processing, we obtained the time courses of the scans of the whole brain. Then, the whole brain was divided into 90 brain regions according to the Anatomical Automatic Labelling (AAL) parcellation atlas [32]. Each brain region was calculated as an average time series and then correlated with the time series of all other brain regions to obtain a 90*90 correlation matrix.

#### ReHo

Regional homogeneity (ReHo) was calculated in each region by REST software (http://www.restfmri.net/). Each voxel and its adjacent voxels were selected to calculate the ReHo in the same time series, which was then assigned to the initial voxel. The ReHo values were standardized and spatial smoothed. Statistical Parametric Mapping 8 (SPM8) [33] was used to perform two-sample T-tests with whole-brain and threshold using FWER at *p* <0.05.

#### Node betweenness centrality

According to graph theory, the node betweenness centrality of each brain region was calculated based on functional connectivity. Node betweenness centrality is the fraction of all shortest paths in a network that contains a given node. In this study, the node betweenness centrality was calculated by the tools implemented in the Brain Connectivity Toolbox (version 2015-01-25) [34].

### Statistical analysis

Two-way ANOVA was used to analyze the effect of smoking and COPD on the attention ability scores of the MoCA scale. Independent-samples T-tests were used to compare age, years of education, smoking index, smoking history, and pulmonary function (FEV1%, FVC %, and FEV1/FVC) between the COPD group and the NonCOPD group, as well as the attention ability scores, smoking index, smoking history, ReHo and node betweenness centrality of brain regions between the COPD-Smoking group and the COPD-Nonsmoking group. The χ^2^ test was used to compare gender between the COPD group and the NonCOPD group. Pearson’s correlation was used to analyze the relationship between the smoking index/smoking history and the ReHo. Kendall’s tau-b correlation analysis was used to analyze the relationship between the ReHo and the attention ability scores, as well as between the smoking index and the attention ability scores since the attention ability scores are rank variables. Bootstrap mediation analysis [35] was used to analyze the mediating effect of ReHo on smoking index and attention ability. Finally, considering the imbalance of sex ratio between smokers and non-smokers, we performed covariance analysis using gender as a covariable in the analysis of attention ability.

## Results

### Among patients with COPD, the smokers had a shorter course of COPD than the non-smokers

There were no significant differences in gender, age, and years of education between the COPD group and the NonCOPD group (Table 1). Independent-samples T-tests showed no significant differences in the smoking index $(t_{40} = 0.44, \;p = 0.665,\; Cohen^{\prime}s\; d = 0.136)$ and the smoking history $\left( {t_{40} = 0.88, \;p = 0.383, \;Cohen^{\prime}s \;d = 0.272} \right)$ between the COPD-Smoking and NonCOPD-Smoking groups.

First, we analyzed the impact of smoking on the course of COPD and found that the course of COPD in the COPD-Nonsmoking group was significantly higher than that in the COPD-Smoking group ($t_{40} = 3.51, \; p = 0.001, \;Cohen^{\prime}s \;d = 1.086$). Smoking occurred earlier than COPD in the COPD-Smoking group ($t_{19} = 17.81, \;p < 0.001, \;Cohen^{\prime}s\; d = 3.982)$. However, independent-samples T-tests showed that there were no significant differences in FEV1% ($t_{40} = - 0.74, \; p = 0.463,\; Cohen^{\prime}s \;d = - 0.229$), FVC% ($t_{40} = 0.81, \;p = 0.424, \;Cohen^{\prime}s \;d = 0.250$) and FEV1/FVC ($t_{40} = - 1.43,\; p = 0.163, \;Cohen^{\prime}s \;d = - 0.442$) between the COPD-Smoking group and COPD-Nonsmoking group, which suggested that COPD caused a consistent lung condition in these two groups.

### Among patients with COPD, the smokers showed less attention impairment than the non-smokers

**Fig. 1:**
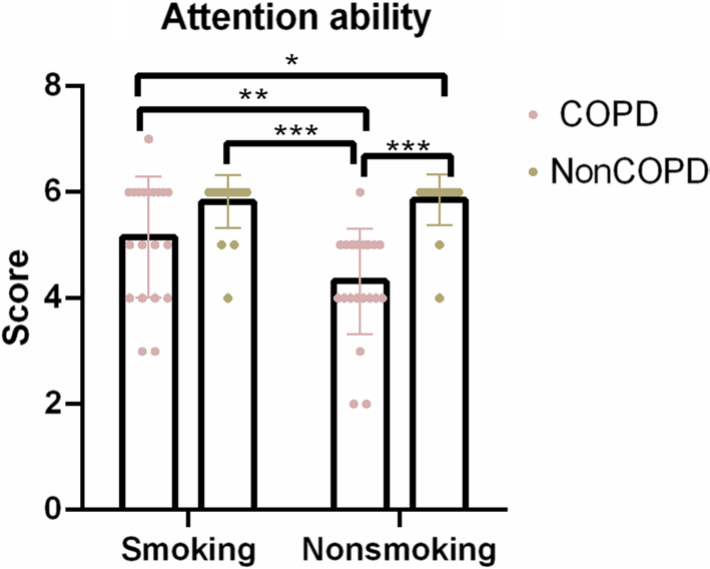
The attention ability scores of the four groups (COPD-Smoking/COPD-Nonsmoking/NonCOPD-Smoking/NonCOPD-NonSmoking) were compared. The *p* values were adjusted by Bonferroni’s correction for multiple comparisons. (*p < 0.05; **p < 0.01; ***p < 0.001)

To understand more about the effect of smoking habit on attention in COPD patients, two-way ANOVA of the attention ability scores was performed and revealed a significant interaction effect (COPD + Non-COPD × Smoking + Nonsmoking) $(F_{{\left( {1,81} \right)}} = 5.91,\;{ }p = 0.017,{ }\;partial\;\eta^{2} = 0.068){ }$ on the attention ability scores and a significant main effect of COPD $(F_{{\left( {1,81} \right)}} = 37.94,\;p < 0.001,\;{ }partial\;{ }\eta^{2} = 0.319)$ and smoking habit on the attention ability scores ($F_{{\left( {1,81} \right)}} = 4.90,\;p = 0.030,\;partial\;\eta^{2} = 0.057)$, which suggest that smokers showed lower impairment of attention ability in COPD patients (Fig. 1, Table 2). In particular, the independent-samples T-test showed that the attention ability scores ($t_{40} = 2.53, \;p = 0.016, \;Cohen^{\prime}s\; d = 0.781$) were higher in the COPD-Smoking group than in the COPD-Nonsmoking group. In addition, the analysis of covariance including sex still showed significant interaction effects (COPD × smoking habit) ($F_{{\left( {1,80} \right)}} = 5.82,\;{ }p = 0.018,{ }\;partial{ }\;\eta^{2} = 0.068).$ The attention ability scores were measured by the MoCA scale.

**Table 2:** Results of cognitive function and pulmonary function assessment (Mean ± SD)

Characteristics	COPD-Smoking (n=20)	COPD-Nonsmoking (n=22)	NonCOPD-Smoking (n=22)	NonCOPD-Nonsmoking (n=21)
Age	68.30 ± 8.12	70.86 ± 7.87	66.45 ± 9.04	70.24 ± 7.62
Education	6.90 ± 4.94	4.73 ± 4.52	6.36 ± 4.01	4.81 ± 3.84
Gender(female/male)	0/20	14/8	0/22	13/8
Attention ability	5.15 ± 1.14	4.32 ± 0.99	5.82 ± 0.50	5.86 ± 0.48
Total score	23.05 ± 3.59	19.73 ± 3.61	26.95 ± 2.40	28.67 ± 2.54
FEV1%	50.30 ± 17.21	54.23 ± 17.08	101.36 ± 9.73	102.95 ± 10.37
FVC%	67.95 ± 16.20	64.05 ± 15.14	90.32 ± 7.23	89.67 ± 6.85
FEV1/FVC	54.30 ± 9.30	58.14 ± 8.16	87.27 ± 5.20	87.00 ± 4.92
COPD history	9.05 ± 7.56	19.36 ± 10.97	None	None

The attention ability was measured by the MoCA scale. FVC FEV1%: forced expiratory volume in one second. FVC%: forced vital capacity

### Among patients with COPD, smoker showed lower ReHo value than the non-smokers

**Fig. 2:**
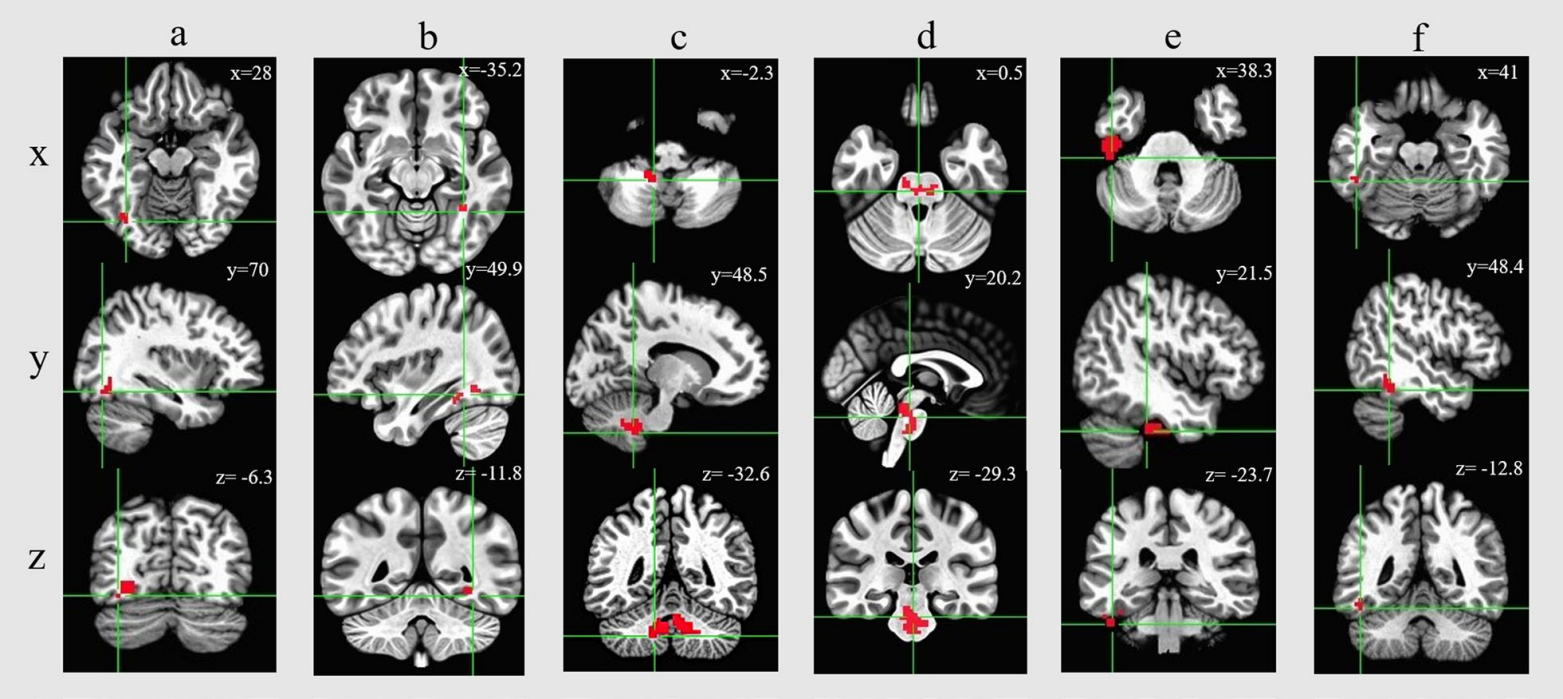
The differences in the ReHo between the COPD and NonCOPD groups are shown in axial, sagittal, and coronal sections. Six clusters were found on whole-brain T-test analysis, and the red areas indicate higher ReHo values. **a** Left fusiform gyrus; **b** Right fusiform gyrus; **c** Left anterior cerebellum; **d** Pons; **e** left inferior temporal gyrus (cluster-1); **f** Left inferior temporal gyrus (cluster-2)

To explore the neural mechanisms of attention ability impairment in COPD, we performed a whole-brain T-test analysis. The results showed that the ReHo of the six clusters in the COPD group, which were located in the bilateral fusiform gyrus, left inferior temporal gyrus, left anterior cerebellar lobe, and pons were lower than those in the NonCOPD group after family wise error correction (FWER) correction (Table 3 and Fig. 2).

**Table 3:** Results of whole brain analysis in ReHo (COPD vs. NonCOPD)

Areas	Cluster size	Talirach corodinate			t	*p*
		x	y	z		
Left fusiform gyrus	55	28	70	−6.3	6.841	0.023
Right fusiform gyrus	42	−35.2	49.9	−11.8	6.383	0.002
Left anterior cerebellum	156	−2.3	48.5	−32.6	5.985	0.019
Pons	148	0.5	20.2	−29.3	6.515	0.016
Left inferior temporal gyrus(cluster-1)	91	38.3	21.5	−23.7	7.974	0.012
Left inferior temporal gyrus(cluster-2)	28	41	48.4	−12.8	6.009	0.001

**Fig. 3:**
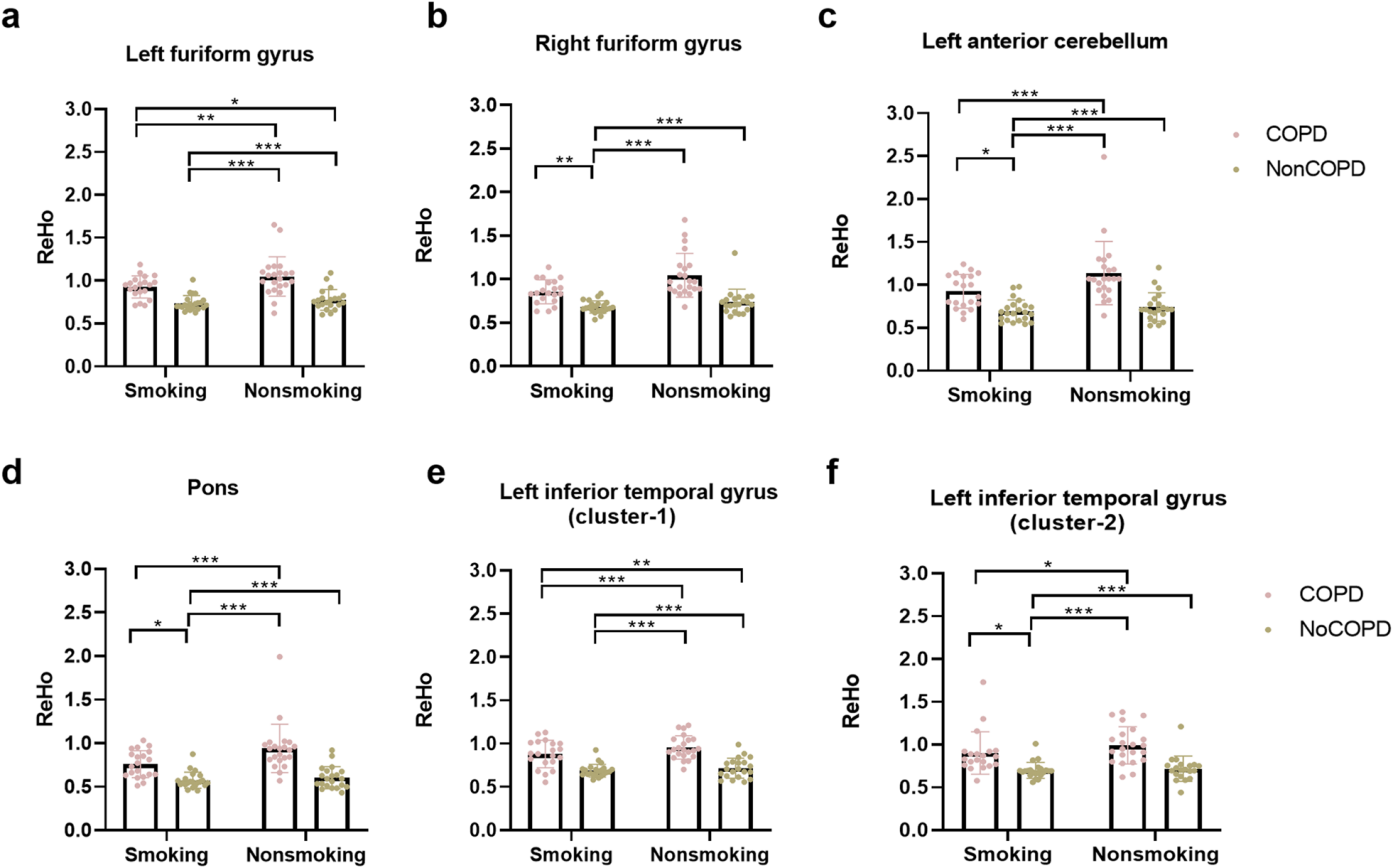
The ReHo values of the four groups (COPD-Smoking/COPD-Nonsmoking/NonCOPD-Smoking/NonCOPD-NonSmoking) were compared in six brain regions. The *p* values were adjusted by Bonferroni’s correction for multiple comparisons. **a** Left fusiform gyrus; **b** Right fusiform gyrus; **c** Left anterior cerebellum; **d** Pons; **e** Left inferior temporal gyrus (cluster-1); **f** Left inferior temporal gyrus (cluster-2). (*p < 0.05; **p < 0.01; ***p < 0.001)

In particular, independent-samples T-tests showed that the ReHo of the left fusiform gyrus $(t_{40} = 2.09, \; p = 0.043,\; Cohen^{\prime}s\; d = 0.646)$, right fusiform gyrus $(t_{40} = 2.96, \; p = 0.005,\; Cohen^{\prime}s \;d = 0.915)$, left anterior cerebellum ($t_{40} = 2.29,\; p = 0.027, \;Cohen^{\prime}s \;d = 0.708$), and pons ($t_{40} = 2.56, \;p = 0.014,\; Cohen^{\prime}s \;d = 0.791$) were all higher in the COPD-Nonsmoking group than in the COPD-Smoking group (Fig. 3).

### ReHo and functional connectivity of the left fusiform gyrus were correlated with attention ability in the COPD groups

Kendall’s tau-b correlation analysis was used to analyze the relationship between the ReHo and the attention ability scores, as well as between the smoking index and the attention ability scores since the attention ability scores are rank variables. The analysis showed that the ReHo of the left fusiform gyrus ($r_{42} = 0.333, \;p = 0.005$), right fusiform gyrus ($r_{42} = 0.259, \;p = 0.030$), and left inferior temporal gyrus ($r_{42} = 0.316, \;p = 0.008$) were associated with the attention ability scores in the COPD groups.

**Fig. 4:**
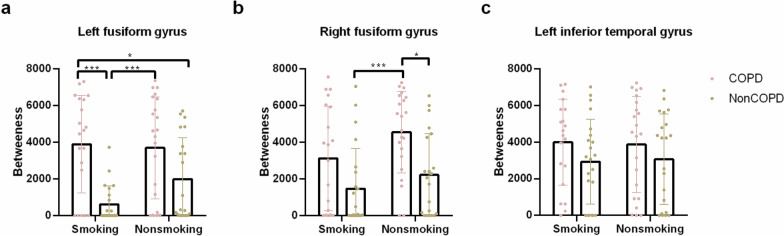
The node betweenness centrality of the four groups (COPD-Smoking/COPD-Nonsmoking/NonCOPD-Smoking/NonCOPD-NonSmoking) was compared in the **a** left fusiform gyrus, **b** right fusiform gyrus, and **c** left inferior temporal gyrus. The *p* value was corrected by Bonferroni’s multiple comparisons test. (*p < 0.05; **p < 0.01; ***p < 0.001)

Two-way ANOVA of node betweenness centrality showed a main effect of smoking in the right fusiform gyrus ($F_{{\left( {1,81} \right)}} = 4.40,{ }\;p = 0.039,{ }\;partial\;\eta^{2} = 0.052$) and a main effect of COPD in the left fusiform gyrus $(F_{{\left( {1,81} \right)}} = 25.39,{ }\;p < 0.001,\;{ }partial\;\eta^{2} = 0.239)$ and right fusiform gyrus $(F_{{\left( {1,81} \right)}} = 14.79,\;p < 0.001,{ }\;partial\;\eta^{2} = { }0.154$). However, there was no significant interaction effect (COPD × smoking) (Fig. 4).

To further understand the role of these three brain regions (left fusiform gyrus, right fusiform gyrus, left inferior temporal gyrus) in attention ability impairment, we analyzed the functional connectivity between them and all other brain regions. Kendall’s tau-b correlation analysis found that the functional connectivity between the left fusiform gyrus and the left pallidum was positively correlated with the attention ability scores ($r_{42} = 0.253, \;p = 0.033$) and total MoCA score ($r_{42} = 0.338, \;p = 0.002$) in the COPD groups.

### Mediating analysis: ReHo of the left fusiform gyrus mediated the influence of smoking on attention ability in the COPD group

**Fig. 5:**
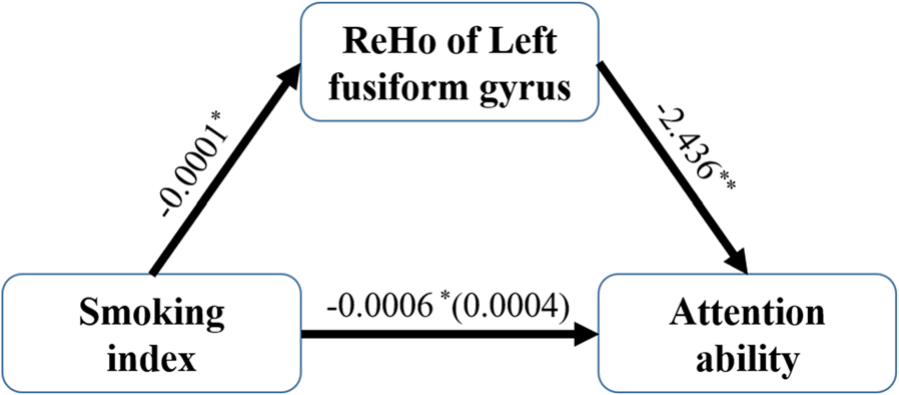
The ReHo of the left fusiform gyrus completely mediates the influence of smoking on attention ability. The regression coefficient is shown in the figure. (*p < 0.05; **p < 0.01)

To understand more about the mechanism of the impact of smoking on the attention ability in COPD patients, we analyzed the relationships between the smoking index and the attention ability scores and the ReHo. First, Kendall’s tau-b correlation analysis found that the smoking index was positively related to the attention ability scores in COPD patients ($r_{42} = 0.336, \;p = 0.030$), including the COPD-Smoking group and COPD-Nonsmoking group. Pearson’s correlation was used to analyze the relationship between smoking index/smoking history and the ReHo, since these were all continuous variables, and found that the smoking index was negatively related to the ReHo of the left fusiform gyrus in COPD patients ($r_{39} = - 0.345,\; p = 0.025$). Mediation analysis revealed that the ReHo of the left fusiform gyrus completely mediated the effect of the smoking index on the attention ability scores in the COPD group $({\text{LLCI}} \sim {\text{ULCI}}: - 0.0002 \sim 0.0011,\;{\text{ BootLLCI}} \sim {\text{BootULCI }}:{ }0.0001 \sim 0.0008$) but not in the NonCOPD group (Fig. 5).

## Discussion

COPD is often comorbid with smoking; however, how smoking modulates behavior and its neural basis in COPD-Smoking comorbidity remains unclear. At the behavioral level, we found that the COPD-Smoking had higher attention ability than the COPD-Nonsmoking. At the neural level, we found that the ReHo of the left fusiform gyrus completely mediated the effect of smoking on attention ability. The founding of this study suggested that smokers showed less impairment of attention ability in COPD-Smoking comorbidity, which is related to the function of the left fusiform gyrus.

Nicotine is not only an important component in cigarettes that causes smoking addiction but also has a protective effect on dopaminergic neurons. Neuronal nicotinic acetylcholine receptors in the brain are more often associated with neuromodulation than the mediation of synaptic transmission. The complex Ca^2+^ response generated by the activation of the nicotinic acetylcholine receptor can transmit information beyond the initial domain and promote the activation of many intracellular signaling pathways. These mechanisms form the basis for the diversity of nicotinic neuron activity in the brain, from the enhancement of learning and memory to addiction and neuroprotection [27]. Thus, the protective mechanism of nicotine may be responsible for less attention impairment in COPD-Smoking comorbidity. Moreover, smoking occurred earlier than COPD in the COPD-Smoking group. Presumably, nicotine may preempt a protective mechanism that buffers cognitive impairment in COPD-Smoking comorbidity.

We found that the ReHo of the left fusiform gyrus mediated the influence of smoking on attention ability, which suggested that it may be the central brain region of the acute effect of smoking. The fusiform gyrus is an integral part of the ventral occipito-temporal conjoint brain region. Previous studies have shown that this region was widely involved in cognitive processes such as face recognition, object position and vocabulary and was associated with visual spatial attention [36]. Thus, the fusiform gyrus is likely a central factor in the effect of smoking on attention ability in COPD-Smoking comorbidity. In addition, whole-brain analysis also found differences in left inferior gyrus, left anterior cerebellum and pons between COPD and non-COPD groups. Previous studies have shown that inferior gyrus may be related to the phonological information storage, behavior chosen or attention modulation [37]. And the neural network involved in the age-related decrements in memory and attention included IFG and the cerebellum [38], which meant these brain regions may be the key factor in cognitive impairment for COPD patients.

We were also concerned about other cognitive functions in the MoCA scale, including visuospatial execution ability, naming ability, language ability, abstraction ability and delayed recall ability. We found that these functions were all impaired in COPD patients (Additional file 1: Table S1). This finding is consistent with previous studies [39–41]. Specifically, only visuospatial execution ability and abstraction ability were significantly worse in the COPD-Nonsmoking than in the COPD-Smoking (Additional file 1: Figure S1 and Additional file 1: Table S4), but no mediation effect was found. In addition, researchers have found that smoking also harms cognitive functions independently, such as memory [42] and execution ability [43]. In this study, we also found that delayed recall ability was worse in the NonCOPD-Smoking group than in the NonCOPD-Nonsmoking group (Additional file 1: Figure S1 and Additional file 1: Table S2). Overall, the independent effects of smoking and COPD on other cognitive functions are consistent with previous studies. The role of smoking in the impairment of these cognitive functions in COPD patients may be similar to that in the impairment of attention ability, but the neural mechanisms remain unclear and deserve further study.

Research suggested that smoking is a major risk factor for COPD, in addition to occupational exposure and indoor and outdoor air pollution. The primary modifiable risk factors to prevent COPD are represented by smoking cessation as well as avoiding air pollution or occupational exposure [44]. Attention deficits were often reported by COPD patients [45]. The worse airflow limitation and old age were associated with attention deficits in COPD patients [14]. Smoking cessation is the only evidence-based intervention that reduces lung function decline in COPD [46]. FEV1 initially increases after smoking cessation [47, 48]. Smoking cessation interventions are usually a combination of behavioral and pharmacological treatment. The behavioral treatment includes behavioral and cognitive therapies (BCT). The standard pharmacological treatments include nicotine replacement therapy (NRT) (e.g., nicotine gum, nicotine inhalers), nicotine replacement partial agonists (NRPAs) (e.g., Varenicline), and antidepressants for smoking cessation (ADs) (e.g., bupropion). The bupropion aid long-term smoking cessation and efficacy are similar to that of NRT [49]. The varenicline increased the chances of successful long-term smoking cessation compared with bupropion [50]. Comparison of NRT and varenicline suggested a minor benefit of varenicline tending towards equivalency [50]. Interventions that combine behavioral and pharmacological treatments are even more successful [51]. And in our study, we found the protective mechanism of nicotine may be responsible for the less attention impairment in COPD*-*smoking comorbidity. Therefore, NRT combined BCT may be preferred for smoking cessation treatment of COPD in the future.

Although we aimed to ensure a high-quality study, there were still some limitations. First, there is an imbalance of sex ratio between smokers and non-smokers, which is due to the very low prevalence (2.7%) of female Chinese smokers and the possible influence of the menstrual cycle phase on smoking cue reactivity and cigarette craving [52]. Trying to eliminate the influence of sex on the results of this study, we performed covariance analysis on the main results using sex as a covariable, and the results were consistent (see Results). Second, the subjects in this study were on average old (mean 68.96 years old) and with low education (mean 5.68 years), which may have led to an over-evaluation of the degree of cognitive decline. Third, the MOCA test provided relatively less information about cognitive performance. Other measures of attention function should be included in the future study, like the dot-probe task [53] and visual search task[54]. Fourth, the sample size of this study was too small to stratify disease according to severity. It is hoped that in future work, more female smokers, more accurate indicators, and larger sample sizes can be included, and stratified studies can be conducted according to the severity of the disease or other factors. Last, we did not have detailed information on exposure to other sources of smoke in the COPD-Nonsmoking group.

## Conclusions

This study revealed the effect of smoking on attention ability in COPD-Smoking comorbidity. Smokers showed less attention impairment in COPD-Smoking comorbidity, which is mediated by the ReHo of the fusiform gyrus. The results support the acute effect of smoking on behaviors in COPD*-*Smoking comorbidity. While previous studies have tended to exclude smoking from their focus on COPD, the findings in this paper are the first to reveal the positive aspect of smoking in COPD, suggesting an important role of smoking in COPD-Smoking comorbidity and potentially in other diseases.

## Supplementary Information

**Additional file 1:** Online Supplement.

## Data Availability

Additional material and all other data for this study can be available from the corresponding authors with reasonable request.

## Abbreviations

COPD: Chronic obstructive pulmonary disease
ReHo: Regional Homogeneity
fMRI: Functional magnetic resonance imaging
MoCA: Montreal cognitive assessment
LG: Lingual gyrus
SMA: Supplementary motor area
PCL: Paracentral lobules
ROI: Regions of interest
FVC: Forced vital capacity
FOV: Field of view
SE-EPI: Spin-echo E-Planar imaging
TR/TE: Repetition time/echo time
MNI: Montreal Neurological Institute
WM: White matter
CSF: Cerebrospinal fluid
FWHM: Full-width-half-maximum
AAL: Anatomical Automatic Labelling
FWER: Family wise error correction
IFG: Inferior frontal gyrus
BCT: Behavioral and cognitive therapy
NRT: Nicotine replacement therapy
NRPAs: Nicotine replacement partial agonists
ADs: Antidepressants
