# Supplementary material for: Smoking related attention alteration in chronic obstructive pulmonary disease-smoking comorbidity
Source: BMC Pulm Med. 2022 May 6;22:182. doi: 10.1186/s12890-022-01964-6 (PMC9078025; doi:10.1186/s12890-022-01964-6)
Supplement: Supplementary file 1 — Additional file 1: Online Supplement. [file 12890_2022_1964_MOESM1_ESM.docx]

**SUPPORTING INFORMATION**

**SUPPLEMENTARY MATERIAL**

***Cognitive functions in the MoCA scale***

We also explored the impact of COPD on other cognitive functions in the MoCA. The scores of visuospatial execution ability, naming ability, language ability, abstraction ability, delayed recall and total score were all significantly lower in the COPD group than in the NonCOPD group (Table S1). However, all the participants scored full marks for orientation ability.

**Table S1.** Results of Cognitive function in COPD and NonCOPD group

| **Characteristics** | **COPD(**n=42)  Mean±SD | **NonCOPD(**n=43)  Mean±SD | **p-value** | **t-value**  **df=83** |
| --- | --- | --- | --- | --- |
| **Visuospatial executive** | 2.14±1.56 | 3.72±1.14 | <0.001 | 5.34 |
| **Naming** | 2.55±0.59 | 2.98±0.15 | <0.001 | 4.59 |
| **Attention** | 4.71±1.13 | 5.83±0.48 | <0.001 | 5.99 |
| **Language** | 2.45±0.63 | 2.83±0.43 | 0.002 | 3.28 |
| **Abstraction** | 0.88±0.71 | 1.79±0.46 | <0.001 | 7.03 |
| **Delayed recall** | 1.55±1.91 | 3.77±1.27 | <0.001 | 8.30 |
| **Total score** | 21.31±3.94 | 27.79±2.59 | <0.001 | 8.99 |

Specifically, only visuospatial execution ability and abstraction ability were significantly worse in COPD-Nonsmoking than in COPD-Smoking (Figure S1 and Table S2), but no mediation effect was found. In addition, we also found that delayed recall ability was worse in the NonCOPD-Smoking group than in the NonCOPD-Nonsmoking group (Figure S1).

**Figure S1**. The cognitive function of the four groups

**Figure S1**. The cognitive function of the four groups (COPD-Smoking/COPD-Nonsmoking/NonCOP-Smoking/NonCOPD-NonSmoking) was compared. The p values were adjusted by Bonferroni’s correction for multiple comparisons.

**Table S2** Results of independent sample T test in MoCA score (COPD-Smoking vs. COPD-Nonsmoking).

|  | t-value | p-value | df | Cohens’d | LLCI | ULCI |
| --- | --- | --- | --- | --- | --- | --- |
| **Visuospatial executive ^*^** | 2.572 | 0.014 | 40 | 0.397 | 0.25 | 2.07 |
| **Naming** | 1.620 | 0.113 | 40 | 0.250 | -0.07 | 0.65 |
| **Attention^*^** | 2.529 | 0.016 | 40 | 0.391 | 0.17 | 1.50 |
| **Language** | 0.461 | 0.647 | 40 | 0.071 | -0.31 | 0.49 |
| **Abstraction^*^** | 2.503 | 0.017 | 40 | 0.387 | 0.10 | 0.93 |
| **Delayed recall** | 1.595 | 0.119 | 40 | 0.246 | -0.15 | 1.31 |
| **Total score^*^** | 2.985 | 0.005 | 40 | 0.461 | 1.07 | 5.57 |

We also analysed the impact of smoking on other cognitive functions in the MoCA. Two-way ANOVA of the MoCA score found significant interaction effect in abstraction ability, delayed recall ability and total score,a significant main effect of COPD and smoking on the visuospatial execution ability, naming ability, language ability, abstraction ability, delayed recall ability and total scores, a significant main effect of smoking on visuospatial execution ability(Table S3 and Figure S1).

**Table S3** Results of Two-way ANOVA analysis in MoCA score.

|  | effect | Mean squre | F-value | p-value | LLCI | ULCI |
| --- | --- | --- | --- | --- | --- | --- |
| **Visuospatial**  **executive** | COPD^***^  Smoking^*^  Intercation | 50.92  8.49  5.88 | 29.55  4.93  3.41 | <0.001  0.029  0.068 | -2.12  0.07  -0.08 | -0.98  1.20  2.19 |
| **Naming** | COPD^***^  Smoking  Intercation | 3.79  0.32  0.60 | 21.22  1.79  3.36 | <0.001  0.185  0.071 | -0.61  -0.06  -0.03 | -0.24  0.31  0.70 |
| **Attention** | COPD^***^  Smoking^*^  Intercation | 25.84  3.33  4.02 | 37.94  4.90  5.90 | <0.001  0.030  0.017 | -1.46  0.04  0.16 | -0.75  0.75  1.58 |
| **Language** | COPD^**^  Smoking  Intercation | 3.17  0.27  0.89 | 11.11  0.96  3.11 | 0.001  0.330  0.081 | -0.62  -0.34  -0.87 | -0.16  0.12  0.05 |
| **Abstraction** | COPD^***^  Smoking  Intercation^*^ | 17.15  0.78  2.20 | 52.27  2.38  6.70 | <0.001  0.127  0.012 | -1.15  -0.06  -1.14 | -0.65  0.44  -0.15 |
| **Delayed recall** | COPD^***^  Smoking  Intercation^***^ | 104.80  3.46  20.43 | 83.23  2.75  16.23 | <0.001  0.101  <0.001 | -2.71  -0.89  -2.93 | -1.74  0.08  -0.99 |
| **Total score** | COPD^***^  Smoking  Intercation^***^ | 875.00  13.76  134.50 | 92.18  1.45  14.16 | <0.001  0.232  <0.001 | -7.75  -0.53  2.37 | -5.09  2.14  7.70 |

In particular, the independent samples T test showed that the visuospatial execution ability ($t_{40}= 2.57, p=0.014, Cohen^{'}s d=0.795$), abstraction ability ($t_{40}= 2.50, p=0.017, Cohen^{'}s d=0.773$) and total scores ($t_{40}= 2.99, p=0.005, Cohen^{'}s d=0.922$) were all higher in the COPD-Smoking group than in the COPD-Nonsmoking group (Table S4 and Table S2).

**Table S4.** Results of Cognitive function and Pulmonary function assessment.

| **Characteristics** | **COPD-Smoking**(n=20) | **COPD-Nonsmoking**(n=22) | **NonCOPD-Smoking**(n=22) | **NonCOPD-Nonsmoking**(n=21) |
| --- | --- | --- | --- | --- |
| **Visuospatial**  **executive** | 2.75±1.29 | 1.59±1.59 | 3.77±1.34 | 3.67±0.91 |
| **Naming** | 2.70±0.47 | 2.41±0.67 | 2.95±0.21 | 3.00±0.00 |
| **Attention** | 5.15±1.14 | 4.32±0.99 | 5.82±0.50 | 5.86±0.48 |
| **Language** | 2.50±0.69 | 2.41±0.59 | 2.68±0.57 | 3.00±000 |
| **Abstraction** | 1.15±0.67 | 0.64±0.66 | 1.73±0.46 | 1.86±0.48 |
| **Delay recall** | 1.85±1.18 | 1.27±1.16 | 3.09±0.87 | 4.48±1.25 |
| **Total score** | 23.05±3.59 | 19.73±3.61 | 26.95±2.40 | 28.67±2.54 |
